# Incorporating Mixed Reality for Knowledge Retention in Physiology, Anatomy, Pathology, and Pharmacology Interdisciplinary Education: A Randomized Controlled Trial

**DOI:** 10.1007/s40670-022-01635-5

**Published:** 2022-09-23

**Authors:** Vineesha Veer, Charlotte Phelps, Christian Moro

**Affiliations:** Faculty of Health Sciences and Medicine, Bond, University, Gold Coast, Australia

**Keywords:** Disease education, HoloLens, Learning, Teaching, Educational technology

## Abstract

Disease education is a fundamental component in health science and medicine curricula, as it prepares students for their progression into health profession careers. However, this requires an ability to integrate concepts across multiple disciplines. Technology-enhanced interventions may bridge this gap, and this study assessed the effectiveness of a textbook-style or a three-dimensional mixed reality (MR, a hybrid of augmented and virtual reality) HoloLens resource for student learning and knowledge retention using asthma as a model of disease. Sixty-seven first-year undergraduate health science and medical students were randomized into two groups to complete a lesson on the physiology, anatomy, pathology, and pharmacology of asthma, delivered through either a textbook-style (*n* = 34) or MR (*n* = 33) resource. Participants took part in the study in small groups and completed the intervention and surveys in separate areas of a large laboratory space. A pre-test prior to the lesson included multiple-choice questions, with the post-test having additional multiple-choice questions to assess learning. A follow-up test to assess retention was performed two weeks later. Pre- and post-test scores revealed increased learning across both the textbook (*p* = 0.001) and MR (*p* = 0.05) interventions, although higher test results were obtained by those using the textbook-style resource (*p* < 0.05). There was no difference between groups in knowledge retention scores. Although the textbook-style resource was more effective for increasing test results, participants perceived MR as more favorable, highlighting the experience as enjoyable and useful. This study presents MR as an option for integration in cases where educators wish to enhance student enjoyment of the learning experience. However, the results suggest that traditional text-based resources persist as a fundamental delivery mode within a modern curriculum.

## Introduction

There is great value in introducing interdisciplinary education into a health professions course. This exposes students to a range of expert opinions and provides a greater breadth of knowledge. This is particularly important when teaching about disease, where there is often a multifactorial impact on body systems [1]. Learning about diseases presents one of the most challenging concepts in medical education, as it often requires an integration of many disciplines to fully comprehend the underlying mechanisms and treatments [2–4]. In addition, for effective learning, teaching should be focused on interventions that encourage knowledge retention, as many students will be expected to have retained a robust knowledge of diseases in their future health professions careers [5–7].

Within a health professions program, across individual science disciplines, the most common teaching method sits as lecture-based delivery [8, 9]. However, there are great benefits in integrating interdisciplinary teaching practices [10, 1], and this is a requirement when teaching about disease. For example, one common disease taught in medical and health science programs is asthma. An integrated knowledge of anatomy, physiology, pathology, pharmacology, and more is required to fully comprehend the disorder, as well as the management and treatment options. While the primary method of teaching asthma is through educator-centered programs [11], it can be challenging for students to grasp overarching interdisciplinary concepts from didactic and passive-learning approaches [12].

Ensuring educational interventions develop long-term learning so that students can recall at a later date is paramount in health science and medicine. In clinical programs, when students partake in ‘cramming sessions’ prior to an assessment, although their results may be sound at the time, this practice appears to develop only fleeting knowledge with a significant decline in assessment results after three months [13]. It is well understood that active learning approaches enhance knowledge retention and recall at later dates [14], commonly after at least two weeks [15–17]. Technology-enhanced supplementary tools, such as mixed reality, may be able to bridge this gap, encouraging an experiential and hands-on way to learn across multiple disciplines [18, 19]. Mixed reality, a hybrid of augmented and virtual reality, is an integration and interaction of both real-world and digital environments [20]. One such example is the Microsoft HoloLens, a mixed reality, head-mounted smart glasses device that produces virtual three-dimensional (3D) virtual holograms in the real world (Microsoft Corporation, Redmond, WA, USA). The HoloLens is a unique teaching device due to its ability to utilize both augmented and virtual reality concepts to present organs and lesson content in true 3D, an important consideration when learning about disease [21, 22].

### Theoretical Rationale

The theoretical background informing this study stems from the fact that unlike the provision of specific content within a set lecture, to fully comprehend a disease, students require integrations of a number of different concepts from a range of disciplines (e.g., physiology, anatomy, immunology, and pharmacology). Mixed reality allows student-directed (e.g., pausing the audio, moving the model, removing layers when desired) learning [23], which may assist in mitigating the intrinsic cognitive load [24]. In addition, the student-centered mode of experiential learning, and the enhanced interactivity provided from mixed reality delivery (e.g., dissecting layers, manipulating the 3D model), allows for an experiential and constructivist approach to learning [25] that may encourage long-term retention of acquired knowledge [12, 26–28]. As such, this study was guided by the research question: *with its multidisciplinary requirements, can disease education be effectively delivered by mixed reality, and does the approach enhance learning and knowledge retention?*

## Materials and Methods

### Study Setting and Participants

First-year undergraduate students (*n* = 160) from the Faculty of Health Sciences and Medicine at an Australian University, with no prior formal knowledge of asthma, were eligible to participate in this randomized controlled trial. Enrolled students included those in a biomedical science, health science, exercise science, and medical program. Although enrolled in different programs, the content taught was the same, and neither cohort had been exposed to any prior asthma education. Participants were recruited through verbal communication after lectures and convenience sampling was used based on participant availability. Sixty-seven participants volunteered to participate, and after signing an informed consent form, participants were randomized into two respective groups: a textbook-style written resource group (*n* = 34) and a mixed reality group (*n* = 33) to learn about asthma. Allocation concealment was performed by sorting participants into the two groups through the use of an opaque envelope with randomized distributions conducted by a statistician. Following randomization, participants completed a paper-based pre-test questionnaire, which were anonymously coded to pair with the post-test. It was not possible to blind participants from the resource group due to the nature of the mixed reality device; however, all responses were recorded anonymously. Participants recruited provided their email address to contact and send the link for the recall test two weeks after the study. Recall data was collected through an anonymous Qualtrics XM (Qualtrics XM, USA) survey and paired with the respective pre-test and post-test scores. The researchers were unaware of the intervention that was related to the sets of results until final analysis. Ethics for this study was obtained and approved by the University’s Human Research Ethics Committee, and all participants received an explanatory statement prior to participation.

### Development of the Application

**Fig. 1:**
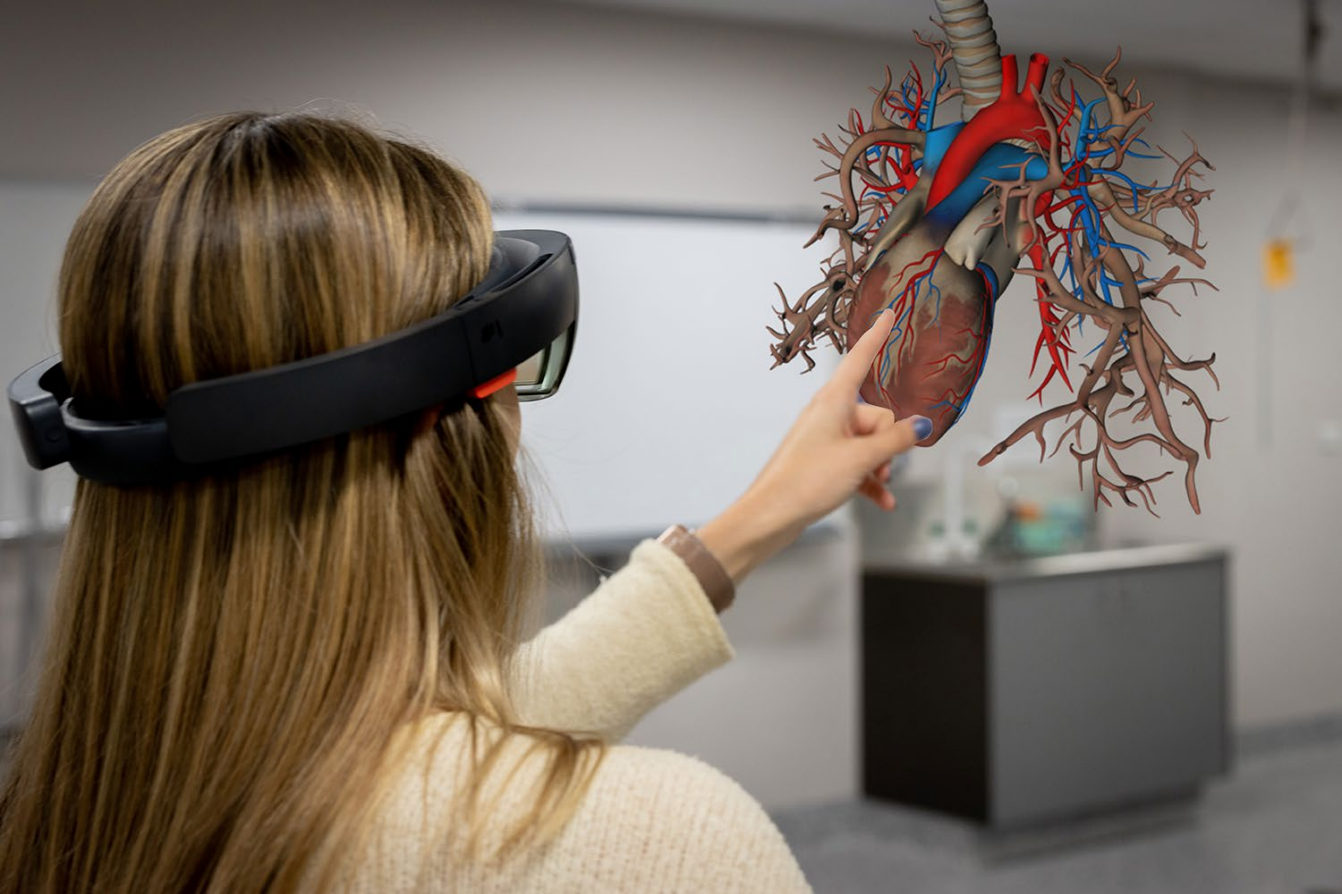
An example of the participant experience when learning through the mixed reality device. The 3D model is visible in front of the user, with hand gestures and voice commands allowing interactivity, while the lesson plays as an audio stream through the headphones

A panel of expert medical educators, clinical doctors, and asthma specialists took part in a series of meetings to discuss which key concepts would be expected within an educational resource incorporating the anatomy, physiology, pathology, and pharmacology of asthma. The decided content involved demonstrating the internal features of the lungs, surrounding organs, muscle structures, and the impact of asthma on the bronchioles. Concepts surrounding the effective pharmaceuticals and the triggering and management of an asthma attack were also included. A 3D model of the lungs and heart was created, edited, labeled, and colorized in-house by the corresponding author using Cinema 4D v21 (Maxon Computer, Friedrichsdorf, Germany). The written text and screenshots of the model were developed into a pamphlet, which was printed and available to participants as the textbook-style resource. For the mixed reality resource, the model was transferred into Unity 3D (Unity Technologies, San Francisco, California, USA), C# coding applied for interactive elements, and exported through Visual Studio v2019 (Microsoft Corporation, Redmond, WA, USA) as a Universal Windows Platform format. All coding, editing, exporting, and digital content in this study was developed in its entirety by the corresponding author. For educators looking to create similar applications on the HoloLens, Microsoft’s “Introduction to Mixed Reality Development” (https://docs.microsoft.com/en-us/learn/modules/intro-to-mixed-reality/) series of online documents contains much of the introductory instructions and content required to commence development. The written text created was read verbatim in the mixed reality lesson (6 min long), allowing for identical content to be provided across both the mixed reality and printed textbook-style resource. Participants could interact with the model using voice (e.g., “dissect,” “remove layer,” “undo”) or hand gesture commands (Fig. 1). To highlight features, the user would hold their finger out and “tap” in the air where the model was displayed. This gesture was detected by the HoloLens and the region became selected with the name displayed in text on the screen. This selected model could then also be dissected by either hand gestures or voice commands to view the underlying anatomy.

### Research Design

Participants in both groups received a brief 2-min lesson on the allocated intervention to ensure an understanding prior to the lesson commencing and to avoid disruption. The mixed reality group was shown how to turn on and off the application and use voice commands and hand gestures to interact with the model. The study utilized pre- and post-tests to assess knowledge gain, as well as an additional post-test administered two weeks after the intervention to measure knowledge retention. Initially, all participants completed a five-question multiple-choice paper-based asthma knowledge pre-test survey to assess baseline knowledge, followed by the commencement of the learning module. After the conclusion of the lesson in the allocated learning module, participants completed a 15-question multiple-choice paper-based post-test, which included five identical questions from the pre-test and ten new questions based on content from the lesson to assess knowledge gained. Examples of questions assessed included “Which part of the respiratory system constricts to cause the symptomology of asthma?” and “What inflammatory chemical in the body causes asthma?”. Such questions were incorporated to reflect the typical assessment format and health science and medical students within their respective degree. Participants also filled out a 10-item Likert scale questionnaire in the post-test which was related to their experience and perceptions of the delivery mode. There was no strict time limit allotted for participants to complete the session; however, each session lasted on average for 15 min. The questions participants answered correctly in the post-test were reassessed two weeks after the learning activity was implemented to assess recall via a non-compulsory online Qualtrics XM survey emailed to participants. When tracked across both health sciences [29] and clinical education [30] programs, students were more likely to take advantage of digital learning resources within the final two weeks prior to the semester’s examination. This timeframe was also commensurate with other retention-based studies in the literature [16].

### Reliability and Validity

To validate the survey questions employed in the study questionnaire, a committee of five academics with experience teaching first-year health science and medical students was established. This expert committee evaluated the face value of the survey and established the validity of the questions. Each item was assessed for clarity, relevance, format, simplicity, grammatical construction, and comprehensibility. There was no training required for participants who completed the survey, which was seen as straightforward and simple to understand. No participants had any queries or questions regarding the survey questions after it had commenced. Questions were assessed for reliability using SPSS v26 (IBM Corporation, Armonk, NY). From this assessment, all questions (25 items) were deemed to have good internal consistency based on the Cronbach alpha value (*α* = 0.866).

### Data Analysis

After successfully testing for the appropriateness of normality, homogeneity of regression slopes, homogeneity of variance, and linearity, a one-way analysis of covariance (ANCOVA) test was applied to determine whether there was a statistical difference between modes of delivery. The statistical software SPSS v26 was used for all analyses. For pre-post testing (five questions) within the same group, a Student’s paired one-tailed *t* test was applied to assess the directional hypothesis that learners would acquire knowledge after learning from either the textbook or mixed reality delivery modes. To analyze statistical significance in overall knowledge retention between the two groups after the two-week time period, a Mann–Whitney *U* test was applied. Participant perceptions using the allocated learning module were rated on a five-point Likert scale (*1*, *strongly disagree*, to *5*, *strongly agree*), where higher scores signified a positive perception using the intervention. A Student’s two-tailed unpaired *t* test was used to analyze participant perceptions. For all statistical tests, *p* < 0.05 was considered statistically significant. Data was processed into figures using GraphPad Prism v8 (GraphPad Software, San Diego, CA, USA).

## Results

A total of 67 participants were included in the final analysis for learning in this study, with 42 participants also being reassessed for knowledge retention two weeks later. No participants were excluded from final analysis. Participants included first-year students from an undergraduate health science or medical program, including 22 male (40%) and 45 female (60%) participants aged ≥ 17 years.

### Knowledge Test Scores

All participants completed the pre-test containing five multiple-choice questions. There was no significant difference for pre-test scores between groups (*p* = NSD, Student’s two-tailed unpaired *t* test), demonstrating a consistent level of background knowledge. Out of the five marks attainable in the pre-test, participants achieved scores (mean ± SEM) of 3.82 ± 0.92 (*n* = 34) in the textbook-style group and 3.82 ± 1.12 (*n* = 33) in the mixed reality group. These same five questions were again asked immediately after the lesson, with participant scores increasing to 4.65 for the textbook-style group (*p* = 0.05) and 4.12 for the mixed reality group (*p* = 0.01, Student’s two-tailed paired *t* test). The overall post-test assessment was out of 15 marks. Participants recorded post-test scores of 13.06 ± 1.79 in the textbook-style group and 11.82 ± 1.85 in the mixed reality group (*p* = 0.011 between the groups, Student’s two-tailed unpaired *t* test).

An ANCOVA was used to examine the post-test scores (out of 15) between the textbook-style and mixed reality groups. As the results for the pre-test (/5) may impact the overall score, this was measured and included in the analysis as a covariate. Before interpreting the outcome of the ANCOVA, the variables were checked for normality using normal Q-Q plots and the Shapiro–Wilk test and assessed to be approximately normal. This was also supported by the skewness and kurtosis statistics being close to zero and a reasonably bell-shaped histogram. In addition, Levene’s test was statistically non-significant, indicating that the assumption of homogeneity of variance had not been violated, F(1, 63) = 0.09, *p* = 0.77. The pre-test covariate was significantly related to the overall post-test F(1, 62) = 64.67, *p* = 0.035. The ANCOVA indicated that, after accounting for the pre-test score covariate, the results for the post-test were statistically significant, F(1, 62) = 6.87, *p* = 0.011, partial η2 = 0.10. Post hoc testing revealed that participants in the textbook-style group obtained higher post-test scores (/15) than the mixed reality group.

### Knowledge Retention Scores After Two Weeks

A total of 42 participants returned to complete the voluntary retention test two weeks after the initial session using the learning modules. This consisted of 22 participants in the textbook-style group and 20 participants in the mixed reality group. From questions assessed in the post-test, after two weeks, overall scores reduced by 1.73 for the textbook-style group and 0.9 for the mixed reality group. No significant difference was observed from either group after two weeks (*p* = NSD, Mann–Whitney *U* test).

### Participant Perceptions

**Fig. 2:**
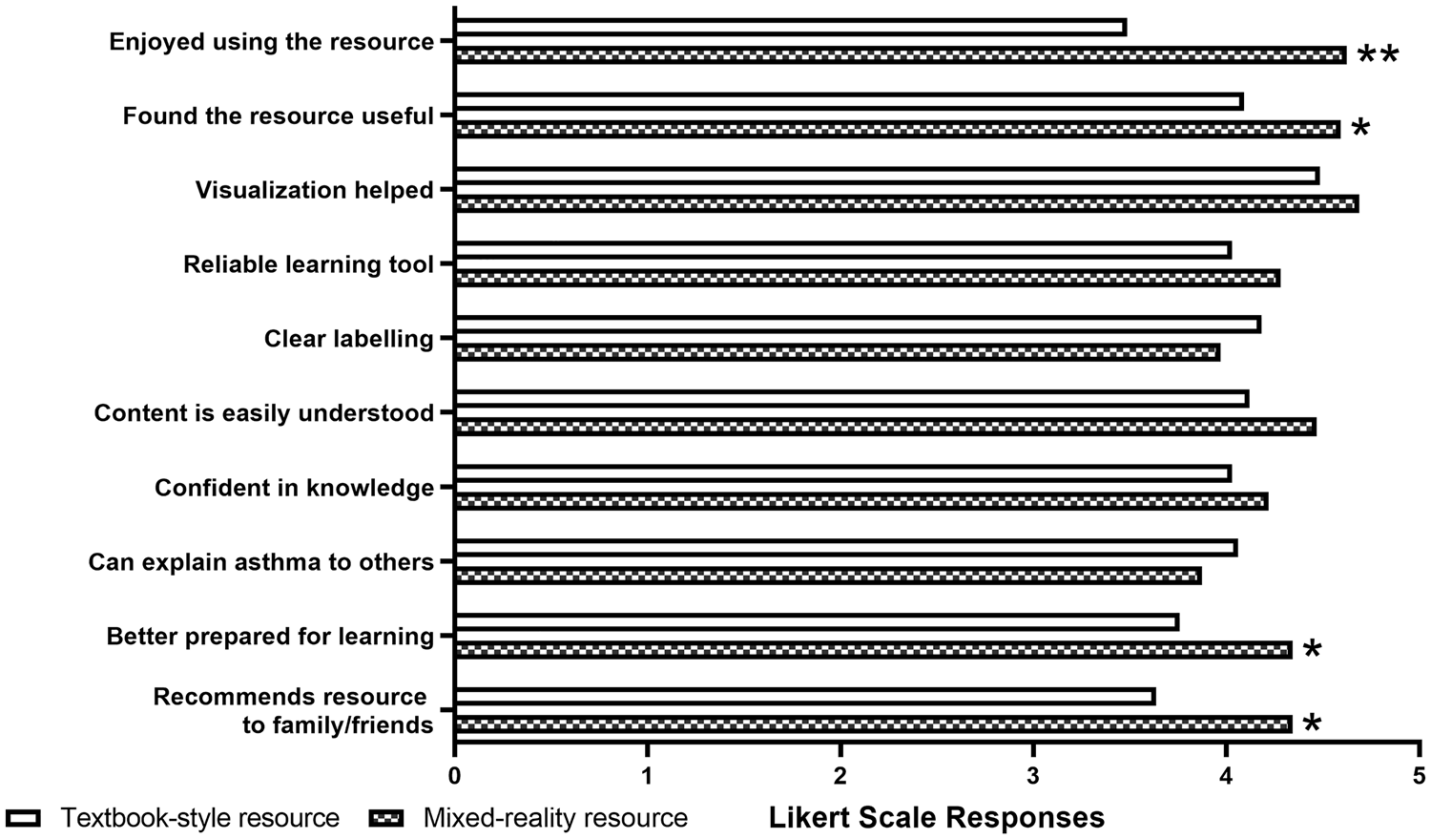
Likert scale responses of participant perceptions from the textbook-style group (*n* = 34) and mixed reality group (*n* = 33), reported as mean $\pm$ SD. Responses marked from 1 (*strongly disagree*) to 5 (strongly agree).*p < 0.01, **p < 0.001

Participants responded to a five-item Likert scale survey regarding their overall perceptions of their allocated learning intervention. Overall, participants rated their learning experience highly, regardless of the delivery mode (Fig. 2), with more from the mixed reality group reporting positive perceptions of this resource. Participants in the mixed reality group reported this delivery mode to be more enjoyable and useful for learning. In addition, it was reported that the content prepared them better for future asthma education sessions and that they would recommend it for learning to friends and family (*p* < 0.01 for all, Students unpaired two-tailed *t* test).

Optional written feedback was also provided from participants in both the textbook-style (*n* = 7) and mixed reality (*n* = 14) groups after using the allocated intervention. Although this was not substantial enough to thematically analyze, there was a general focus on the tedious nature of the textbook resource and the innovative nature of the mixed reality device (Table 1).

**Table 1:** Representative written comments from participants in the textbook-style and mixed reality groups

**Textbook-style group**
“For a person looking at the respiratory system for the first time, it is hard to remember specific things like numbers and names, with textbook style learning.”
“Bolded text helped me remember certain words and the pictures helped.”
“I don’t think I’ll be able to remember much. Kind of goes in briefly but after short time it will go out.”
“Very well written and easy to read. Appreciate the diagrams very helpful.”
**Mixed reality group**
“Really fun, innovative and informative! The visually 3D presentation, in conjunction with the audio, was an enriching experience.”
“I was a little unsure if I was looking at the right areas of the image, but completely loved the experience. It’s amazing way to focus and be engrossed in the subject. I would have preferred to be in a room by myself, and given instructions right before starting, with no other interruptions. Nonetheless, it was so much fun, thank you.”
“Great experience, however head piece was very uncomfortable.”
“This is an excellent learning tool; the ability to actively dissect and remove layers allows for greater to learn systems and pathology in depth. HoloLens did cause some eye- strain as well.”

## Discussion

There was some success obtained from the introduction of mixed reality to deliver disease education in an undergraduate health science and medicine course. This is of particular interest, as although it is increasingly common for technology-enhanced resources to be implemented within curricula in order to enhance learning [19], it is not always clear which of the various choices is optimal. The potential for mixed reality to stimulate active and experiential learning is a step in the right direction when introducing complex, multidisciplinary curricula to students [31]. Additionally, any impact on long-term retention is important when training future health professionals, as an enhanced understanding of challenging concepts leads to improved long-term retrieval of knowledge, which is heavily relied on when encountering complex cases [32, 33]. In our study, both the mixed reality and textbook-style resources were effective for knowledge acquisition regarding asthma, and both group participants performed well in the post-intervention assessment. Nonetheless, it is of interest to compare between these two delivery modes to see which is optimal in multidisciplinary disease education.

### Participant Learning

Although the pre-post test scores significantly increased for both groups, commensurate with past research into mixed reality education [34], in our study, there was a higher average increase for the textbook-style group, potentially indicating increased effectiveness for traditional textbook-style learning. This suggests that although learning did occur, perhaps users in the mixed reality group were distracted by the technology [35], the novelty of the device, or the additional requirements to learn hand gestures, vocal commands, and other means to interact with the content [36]. The mixed reality HoloLens device has shown promise in prior literature, where it was helpful towards guiding students through the processes involved in catheter placements [37]. In this way, mixed reality may have potential for use beyond simply learning content, extending into actual skill development.

Participants using the mixed reality resource learnt through a three-dimensional representation of the content, while the participants using the textbook-style resource learnt through two-dimensional (2D) diagrams. This may present a confounding limitation, as the literature suggests that 3D learning itself can be helpful in the spatial understanding of a model’s general anatomy [38]. As the printed questionnaire used in this study design was limited to 2D illustrations, students who used the mixed reality resource may have found their mode of learning not commensurate to this assessment format [19].

### Participant Retention

This is the first known study assessing the effectiveness of a mixed reality device for learning and retention of interdisciplinary concepts in health science and medicine. The results suggest that both the HoloLens and textbook-style resources are equally effective as educational interventions for stimulating knowledge which is retained for at least two weeks. This consistent ability to enhance retention may be due to the relevance of the topic [39]. As disease education is a highly important concept to learn for a health professions career, the increased student interest may assist with their overall focus and willingness to learn, regardless of the mode of delivery [40].

### Participant Perceptions

Participants preferred the mixed reality resource over the textbook-style resource, aligning with previous literature in various health disciplines [34]. The mixed reality resource was preferable for enjoyability, usefulness, and the perceived preparedness for any future learning surrounding asthma. Although in our study this increased enjoyment did not translate to the test scores, the finding that students enjoyed using mixed reality more may have educational benefits. Student enjoyability from interacting with technology can further increase self-directed learning [41, 42]. In addition, the novelty of mixed reality may be a contributing factor to student enjoyability [35].

The mixed reality resource was also more likely to be recommended to non-student friends or family for learning and instruction, potentially due to the unique visual method of learning which is currently favored by students [43]. The addition of audio learning, instead of text, may be an appealing alternative [44], as written resources can be daunting to learn and large written sections overwhelming. This, coupled with interactive elements and the self-directed approach to the pace of content delivery when using mixed reality, appears to enhance the overall learning experience [45]. Additional advantages of mixed reality include the ability for users to view the surrounding real world, which can minimize adverse events commonly reported in virtual reality, such as dizziness and disorientation [19]. It also allows the use of recalling strategies, such as writing notes or interacting with the educator for further understanding of content, which has been identified as a particular disadvantage of virtual reality, as the purely virtual environment lacks connection to real-world surroundings.

### Limitations and Future Directions

This study incorporated convenience sampling from a single Australian institution, and its relevance to a broader cohort is not clear. In addition, as a novel technology, most participants were unfamiliar with the mixed reality HoloLens device, meaning that there was the potential to be distracted and not fully engaged with the asthma learning module. To accommodate for this, a longer instructional module could be implemented prior to the lesson’s commencement. An additional limitation was that only asthma was used as a teaching example on the basis that it encapsulated various aspects of disease education and that it is highly interdisciplinary and multifactorial in nature. However, it is not clear if this learning would be effective across a range of other disorders. This study defined the timeframe of two weeks for retention testing. Although this is commensurate with previous literature [16], there is little overall consensus on what constitutes an appropriate delay before the assessment of retention. It would be interesting to investigate different ranges, perhaps beyond six months, to genuinely identify if these interventions are useful for facilitating long-term recall of learned concepts. Although the focus was to teach interdisciplinary approaches, the assessment questions remained specific to individual disciplines. In medical programs, this is commonplace as examination questions are often “tagged” to specific areas, learning objectives, or sessions. Moving to short-answer questions or queries where the students can engage multiple disciplines to answer questions would assist to investigate whether the interdisciplinary approach did help with overall comprehension of the content. Finally, it should be mentioned that there is a considerable cost for procuring a mixed reality device, presenting a limitation towards the broad scalability of this technology to use in health science and medical programs.

## Conclusion

Delivering content through both mixed reality and textbook-style modes are suitable for learning, although the textbook-style format resulted in higher test results. However, learning with mixed reality was perceived to be an enhanced learning experience, and more enjoyable for users. The results suggest that when learning is paramount, a textbook-style resource should still be employed as a fundamental teaching tool within health sciences and medical curricula. However, mixed reality resources remain a viable option to supplement learning, with the added benefit of enhancing user enjoyment, as well as the overall learning experience.
